# Transmissibility of the Monkeypox Virus Clades via Respiratory Transmission: Investigation Using the Prairie Dog-Monkeypox Virus Challenge System

**DOI:** 10.1371/journal.pone.0055488

**Published:** 2013-02-07

**Authors:** Christina L. Hutson, Nadia Gallardo-Romero, Darin S. Carroll, Cody Clemmons, Johanna S. Salzer, Tamas Nagy, Christine M. Hughes, Victoria A. Olson, Kevin L. Karem, Inger K. Damon

**Affiliations:** 1 Division of High-Consequence Pathogens and Pathology, Poxvirus and Rabies Branch, Centers for Disease Control and Prevention, National Center for Emerging and Zoonotic Diseases, Atlanta, Georgia, United States of America; 2 Department of Pathology, College of Veterinary Medicine, The University of Georgia, Athens, Georgia, United States of America; 3 Program in Population Biology, Ecology and Evolution, Emory University, Atlanta, Georgia, United States of America; Saint Louis University, United States of America

## Abstract

*Monkeypox virus* (MPXV) is endemic within Africa where it sporadically is reported to cause outbreaks of human disease. In 2003, an outbreak of human MPXV occurred in the US after the importation of infected African rodents. Since the eradication of smallpox (caused by an orthopoxvirus (OPXV) related to MPXV) and cessation of routine smallpox vaccination (with the live OPXV *vaccinia*), there is an increasing population of people susceptible to OPXV diseases. Previous studies have shown that the prairie dog MPXV model is a functional animal model for the study of systemic human OPXV illness. Studies with this model have demonstrated that infected animals are able to transmit the virus to naive animals through multiple routes of exposure causing subsequent infection, but were not able to prove that infected animals could transmit the virus exclusively via the respiratory route. Herein we used the model system to evaluate the hypothesis that the Congo Basin clade of MPXV is more easily transmitted, via respiratory route, than the West African clade. Using a small number of test animals, we show that transmission of viruses from each of the MPXV clade was minimal via respiratory transmission. However, transmissibility of the Congo Basin clade was slightly greater than West African MXPV clade (16.7% and 0% respectively). Based on these findings, respiratory transmission appears to be less efficient than those of previous studies assessing contact as a mechanism of transmission within the prairie dog MPXV animal model.

## Introduction

*Monkeypox virus* (MPXV) belongs to the genus *Orthopoxvirus* (OPXV) of the family *Poxviridae*, and is thus related to *variola virus* (the causative agent of smallpox). Although naturally occurring smallpox has been eradicated, MPXV remains endemic to the rain forests of Central and Western Africa, with reports of sporadic human outbreaks and areas of endemic disease. Since the eradication of smallpox and subsequent cessation of routine smallpox vaccination, there is an increasing population of unvaccinated people that are fully susceptible to OPXV infection, including MPXV [1]. Human zoonotic MPXV infection is believed to most often result from exposure to infected tissues or excreta via close contact, or handling of, infected native African animals [2], [3]. However, the natural reservoir or reservoirs of MPXV are still unknown. Until recent years, human MPXV had only occurred within Africa. The virus caused an outbreak in the United States in 2003 due to importation of infected African rodents, which transmitted virus to pet black-tailed prairie dogs (*Cynomys ludovicianus)*, subsequently causing human disease [4], [5].

Previous studies have characterized two distinct MPXV clades, West African and Congo Basin [6], [7]; the U.S. outbreak was due to an importation of the West African clade MPXV. In humans, West African MPXV is associated with milder disease, no reported mortality (low-level mortality within Africa can not be excluded) and is rarely associated with person to person transmission (never documented as the sole mode of transmission) [8], [9]. In contrast, Congo Basin MPXV causes approximately 10% mortality and appears to transmit more readily between humans; up to six sequential inter-human transmission events have, with supporting laboratory data, been documented [10]. Currently, the cause or causes of the observed differences in rates of transmission between the two clades are not known.

MPXV continues to be an important public health threat both within Africa and can emerge beyond African borders as evidenced by the 2003 U.S. MPXV outbreak. Animal models to study this virus augment our understanding of virus pathogenesis and allow hypothesis generation per human disease. In previous studies, we have shown that the prairie dog MPXV model is an informative model to study this virus, and illness interventions, within an animal host [11]–[13]. Importantly, prairie dogs challenged with MPXV develop disease courses that emulate key features of human monkeypox (and systemic OPXV) disease; notably a protracted incubation period before the development of disseminated skin lesions as well as differences in mortality and morbidity associated post infection with viruses from the two MPXV clades [11]. Subsequent studies with the prairie dog MPXV model showed that these animals are able to transmit the West African MPXV clade animal to animal through bedding/fomite, direct contact, and respiratory droplet/nasal mucous [14]. The finding that prairie dogs are able to transmit MPXV animal to animal expands the potential use of this animal model to evaluate interventions which may both mitigate the disease course as well as prevent disease transmission.

Herein we sought to evaluate the respiratory transmissibility of viruses from each of the MPXV clades between prairie dogs. Studies were designed to test whether the viruses can be transmitted by respiratory transmission alone and to compare transmission rates between the clades. Two animal challenge studies utilized equivalent virus plaque forming unit challenges and equivalent LD_50_ doses of viruses representing the two clades. Initially animals were challenged with 6×10^3^ pfu; for West African MPXV this is 0.05×LD_50_ (n = 4) and for Congo Basin MPXV 1×LD_50_ (n = 4). The initial inoculation dose was formulated based on the need for morbidity with little mortality within challenged animals for each of the MPXV clades. Therefore results from previous studies which utilized a range of MPXV challenge dosages in this animal model were used to calculate the challenge dose [15]. Subsequently a low-dose Congo Basin MPXV challenge (0.1×LD_50_) (n = 4) was done to simulate a LD_50_ similar to that used in the West African MPXV challenge.

## Materials and Methods

### Animals

All animals were handled in strict accordance with good animal practice as defined by the relevant national and/or local animal welfare bodies, and all animal work was approved by the CDC Institutional Animal Care and Use Committee (IACUC) under an approved protocol (2115-211DAMPRAC) issued by CDC IACUC specifically for this study. All sampling procedures were performed under complete inhalation anesthesia by isoflurane; efforts were made to minimize suffering and strict euthanasia criteria were utilized.

Wild-caught, juvenile black-tailed prairie dogs (*Cynomys ludovicianus*) were obtained from Berthoud Colorado. The animals involved were purchased from a vendor which utilized humane live-trapping techniques (wire cage traps) to capture free-living healthy young animals (<1year old). Only animals free from any signs of illness (as determined by a veterinarian) were transported to the Centers for Disease Control and Prevention (CDC) in Atlanta, GA. Once the animals arrived at the CDC, they were quarantined and housed appropriately under an approved CDC IACUC protocol (1718CARPRAC) until the start of the study. At the start of the study, animals were approximately 2–3 years old and had been prescreened by a veterinarian, determined to be in good health status and found negative for the presence of anti-OPXV antibodies. A sterile PIT tag was injected subcutaneously at the base of the neck for animal identification and non-invasive recording of body temperature. In addition to standard prairie dog chow, a highly palatable monkey biscuit was provided once a day as a measure of individual animal appetence. The average starting weight for primary challenged animals in the West African MPXV experiment was 1108 grams (range 962–1211), and the average for naive animals was 1069 grams (range 902–1365). The average starting weight for primary challenged animals in the Congo Basin MPXV experiment was 1100 grams (range 973–1195), and the average for naive animals was 967 grams (range 825–1154). The average starting weight for primary challenged animals in the low-dose Congo Basin MPXV experiment was 952 grams (range 790–1113), and the average for naive animals was 948 grams (range 706–1290).

### Housing and Experimental Design

All animals were housed within an animal Biological Safety Level-3 (ABSL-3) animal room. Three studies (West African MPXV, Congo Basin MPXV, and low-dose Congo Basin MPXV) to evaluate the respiratory transmission potential of the MPXV clades were performed on three separate occasions. For each study, eight large metal rabbit cages with holes on one side (3 sq. feet cage area; 16.438” high X 19.125” wide X 25.875” long) were utilized to individually house eight animals (four challenged, four naive per study). Cages were placed four inches apart and were kept separated by a metal rod. Ventilation holes (1 inch in diameter) from the challenged animals’ cages faced the holed-side from the naive animals’ cages within a Duo-Flow biosafety cabinet with negative directional airflow, similar in design to that illustrated in an influenza animal challenge study with ferrets (supplementary material) [16]. The difference in the current study compared to the influenza study is that in our study both primary and naive animals were single housed. Additionally, our cages had holes in the sides instead of wire-mesh.

### Viruses

The West African MPXV strain (MPXV-USA-2003-044; DQ011153), collected during the 2003 U.S. outbreak [5], [7], and the Congo Basin MPXV strain (MPXV-RCG-2003-358; DQ011154), collected during a 2003 outbreak of MPXV in the Republic of Congo (RCG) [7] were used in this study. The viruses underwent two passages in African green monkey kidney cells (BSC-40) prior to seed pool production; preparations used for animal challenge inoculums were purified via a sucrose cushion. The titers for all virus inoculums were re-titrated for confirmation post inoculation.

### Animal Inoculation

Experimental infection of prairie dogs with the two MPXV strains was done at separate times to reduce the potential for cross-contamination and to facilitate the logistics of sampling animals. Two sets of experiments were initially done to compare the two MPXV clades utilizing a challenge dose of 5×10^3^ pfu. This challenge dose was calculated based on the morbidity observed in the authors’ previous study. A follow-up study was done with a lower dose of Congo Basin MPXV (7×10^2^) to test an equivalent LD_50_ compared to the West African MPXV clade. Stocks of virus were diluted in PBS. Inocula titers were immediately re-confirmed, p.i., by standard plaque assay (as described below). Animals were infected via an intranasal (i.n.) route of inoculation while under general anesthesia using 3–5% isoflurane administered through a veterinary vaporizer. Animals were inoculated with a total volume of 10 ul i.n. (5 ul in each nostril). After administration of virus to an anesthetized animal, the animal was kept in a supine position for approximately 30 seconds to facilitate complete inhalation of the viral challenge. Additionally, 2 animals for each study were mock infected with PBS.

### Observations and Sampling

Post-inoculation, individual animals were observed daily for signs of morbidity, or malaise (inappetence, decreased activity, recumbence with reluctance to move, etc.) and clinical lesions or rash for up to 34- 41 days depending on the progression of disease. Beginning three days p.i. animals were anesthetized twice a week with isoflurane for sampling purposes. Samples and measurements collected included an oral swab (area swabbed included inner cheeks, tongue and the hard palate); blood, weight, temperature and lesion count (if applicable). Between days 14–28 p.i., naive animals had oral swabs taken an additional two times a week. Strict euthanasia criteria were adhered to throughout the study as follows: any animal that became unresponsive to touch, lost 25% or more starting body weight, or accrued a total score of 10 on the following scale was humanely euthanized: decreased activity (2 points); lethargy, unsteady gait, inappetence (3 points each); labored breathing and recumbence (5 points each).

### Necropsy and Tissue Specimen Collection

Necropsies on all animals were performed according to CDC IACUC-approved standards in an ABSL-3 laboratory and utilizing full ABSL-3 PPE. Samples taken during necropsy included: blood, oral swab, tongue, eyelid, gonad, liver, spleen, submandibular lymph nodes/salivary glands, lungs and lesion (if present). Instruments were decontaminated with 5% Microchem and 70% ethanol and allowed to dry between collections of each tissue. Tissue samples were frozen at −70°C prior to further processing. Oral samples were collected with sterile individual swabs and stored frozen without diluent. After animals were sampled each day, serum was immediately separated from whole blood and stored to be processed for serology and clinical chemistry levels (see below). Tissues and samples were subsequently processed and further prepared for DNA analysis, and virus isolation (see below).

### Sample Preparation for PCR and Viral Growth

Sample processing was performed under BSL-2 conditions with BSL-3 work practices. For whole blood samples, 100 ul of EDTA treated blood was used for DNA extraction and the remaining untreated blood was used for tissue culture propagation. 400 ul of sterile PBS was added to each swab collected. The swab extraction tube systems (SETS) (Roche) protocol was used to recover sample from the swab. DNA was extracted from 100 ul of the swab lysate. The remaining swab eluate was used for virus isolation. For tissue preparation, 1 ml aliquots of PBS and SPEX bead (SPEX Sample Prep) were prepared. The PBS/bead aliquot was then poured into a 1 ml tube containing the individually weighed tissue sample. The GenoGrinder 2000 (SPEX Sample Prep) was then used following the manufacturer’s instructions to create a tissue homogenate. 100 ul of the homogenate was then used for DNA extraction. The remaining homogenate was used for virus isolation. The BioRobot EZ-1 Workstation (Qiagen) was used for DNA extraction from all blood, swab and tissue samples using the Tissue Kit. Samples were incubated at 55°C in lysis buffer for an hour to inactivate viable virus particles prior to DNA extraction.

### Real-time PCR Analysis

Samples were tested in duplicate by real-time PCR using forward and reverse primers and probes complimentary to the conserved (OPXV) E9L (DNA polymerase) gene [17]. DNA values were quantified using purified MPXV DNA standard curves (10 fg –1 ng). A sample was considered positive if it produced CT values (in duplicate) of 37 or below for two consecutive sample days.

### Virus-tissue Infectivity

All samples were stored at −70°C until virus isolation was attempted. Previous analyses demonstrated that real-time PCR detection of MPXV DNA is an assay which can detect trace amounts of MPXV DNA in samples which do not contain viable virus [4]. Therefore, specimens were first tested for presence of OPXV DNA by PCR and, if positive, were subsequently evaluated for viable virus by tissue culture propagation. Each positive sample was titrated in duplicate using 10 fold dilutions of swab eluate, whole blood or tissue slurry on BSC-40 cell monolayers, incubated at 35.5°C and 6% CO_2_ for 72 hours, and subsequently stained with crystal violet and 10% formalin to visualize plaques. Titers were expressed as pfu per milliliter (pfu/ml) of blood or swab eluate; or pfu per gram (pfu/g) of tissues.

### Serologic Analysis

Serum was separated from whole blood and transferred to a clean tube and stored at −20C prior to analysis. A modified ELISA was used for analysis of anti-OPXV immunoglobulin types A and G in separated serum as previously described in detail [11]. An OD-COV of 0.05 or greater was considered positive by ELISA.

### Western Blot (WB) Assay

MPXV grown in BSC-40 cells was purified via a sucrose cushion and heat inactivated prior to being used in WB to identify specific immunodominant bands. Additionally, BSC-40 cell lysate was included in each assay as a control to test for non-specific binding of antibodies to cell lysate potentially remaining in semi-purified viral stock. Approximately 20 micrograms of semi-purified MPXV and crude BSC-40 lysate were incubated with Laemmli buffer containing 5% 2-mercaptoethanol and boiled for 5 minutes. Each sample was separated by 4–20% gradient polyacrylamide gel electrophoresis (Ready Gel Tris-HCL) and proteins were transferred to polyvinylidene difluoride membranes (PVDF) (BioRad). Molecular weight standards were run on each gel (Precision Plus Kaleidoscope 1 Kb, BioRad). After the protein transfer, PVDF membranes were blocked for 1–2 hours using 640 mg of dry milk in 160 ml of PBS and 0.1% Tween (PBST). Membranes were then washed with PBST three times for 10 minutes each wash and then membranes were probed with prairie dog serum at a dilution of 1∶500 (naive animals) or 1∶1000 (primary challenged animals) in blocking buffer at 4°C overnight. Next, membranes were washed with PBST three times for 10 minutes each wash and then exposed to Pierce A/G secondary antibody, alkaline phosphatase (AP) conjugate at a dilution of 1∶3000 in blocking buffer for 1–2 hours (Pierce), followed by three washings as described above. Immuno-reactive interactions between rodent serum antibodies and OPXV proteins were identified using chemiluminescence detection (Immun-Star AP substrate, Biorad). Antiserum from prairie dogs with confirmed MPXV infection and from PBS control animals were used as positive and negative controls.

### Blood Chemistry Values

Serum was separated from whole blood and transferred to a clean tube and stored at −20C prior to analysis. The Piccolo blood chemistry analyzer (Abaxis) was utilized to determine the following blood chemistry profiles: sodium (NA), potassium (K), phosphorus (PHOS), glucose (GLU), calcium (CA), blood urea nitrogen (BUN), creatinine (CRE), alkaline phosphatase (ALP), alanine aminotransferase (ALT), amylase (AMY), total bilirubin (TBIL), albumin (ALB), total protein (TP) and globulin (GLOB).

### Pathology

After euthanasia, a necropsy was performed and tissues were taken as described above. Tissues were fixed in 10% neutral-buffered formalin for at least 36 hours and then transferred to 70% ethanol for routine processing and paraffin-embedding. Paraffin-embedded tissues were sectioned at 4 µm and were stained with hematoxylin-eosin (H&E). Histopathological evaluation was performed by a veterinary pathologist.

### Statistical Analyses

The Wilcoxon rank-sum test was utilized to compare the blood chemistry levels for the three animals that died in the high-dose Congo Basin challenge at day 13 post-infection to those values from uninfected animals on day 13. A p-value of ≤0.05 was considered statistically significant.

## Results

For each study, eight cages with holes on one side were utilized to individually cross-house eight animals. Cages were placed four inches apart and were kept separated by a metal rod. One challenged animal was cross-housed from an uninfected animal from the time of inoculation of the challenged animal until study end.

### West African MPXV Clinical Findings and Disease Transmission (Table 1a)

Three of the four animals challenged with West African MPXV (6×10^3^ pfu intranasally administered) developed an infection resulting in illness. The fourth animal never developed symptoms of disease (i.e. skin lesions, immune response, viral shedding). The three primary challenged/infected/ill animals had a typical MPX disease presentation, as we have previously seen and characterized in this model. Characteristic disseminated skin pustules developed on all three animals between days 10–13 and viral DNA in blood was detectable from all three of these animals as was viable virus from oropharyngeal samples. Viral shedding began on day 10 or 13 post infection (p.i.) and lasted until day 17–24; viral loads from the oropharyngeal samples peaked at 2×10^5^–1×10^6^ pfu/mL. Clinical symptoms seen in these three primary challenged animals included skin lesions, crusty noses, dehydration and inappetence (however, weight loss was minimal and comparable to uninfected animals). These three animals developed evidence of MPXV infection via western blot and ELISA. The fourth animal demonstrated no western blot, nor ELISA reactivity to OPXVes post intranasal challenge.

None of the naive animals housed across from the 3 infected, symptomatically ill West African MPXV challenged animals showed any clinical symptoms during the study (inappetance, skin lesions, lethargy, etc.). Testing of oral swabs and blood samples from the naive animals taken during the study were negative for viral DNA and no serologic evidence of anti-*OPXV* reactivity was found by western blot or ELISA at study end (table 1).

**Table 1:** Clinical and Molecular Observations.

	West African MPXV (A)								Congo Basin MPXV (B)								Low Challenge Dose Congo Basin MPXV (C)							
Animal Number	8141	8145	8124	8153	8122	8140	8137	8139	8025†	8142	8027†	8029	8023†	8028	8121	8021†	9125	9116	9034	9024	9049†	9126	9105	9088
Day of Lesion Onset(max lesions)	**13** **(42)**	–	**10** **(8)**	–	**13** **(25)**	–	**–**	–	**10** **(>50)**	–	**10** **(10)**	–	**10** **(15)**	–	**13** **(>50)**	20(15)	**–**	–	**–**	–	**10** **(>50)**	–	**13** **(15)**	–
Day of Initial Antibody Detection	**13**	–	**17**	–	**13**	–	**–**	–	**13**	–	**13**	–	**13**	–	**17**	34*	**–**	–	**–**	–	**13**	–	**13**	–
Western Blot Bands (kD)	**30–39, 62**	–	**30–39, 62**	–	**30–39, 62**	–	**–**	–	**NT**	–	**NT**	–	**NT**	–	**30–39, 62**	NT	**–**	–	**–**	–	**NT**	–	**30–39, 62**	–
Viral DNA Blood	**13–17** **	–	**10–20**	–	**10–20**	–	**–**	–	**3–13**	–	**6–13**	–	**3–13**	–	**10–24**	17**–**34	**–**	–	**–**	–	**6–20**	–	**13–17**	–
Viral DNA oral secretion	**13–24**	–	**10–24**	–	**10–27**	–	**–**	–	**6–13**	–	**6–13**	–	**10–13**	–	**10–34**	17**–**34	**–**	–	**–**	–	**10–20**	–	**10–25**	–
																								
Virus in Oral Secretions	**13–20**	–	**13, 24**	–	**10–17**	–	**–**	–	**6–13**	–	**6–13**	–	**10–13**	–	**10–24**	20**–**34	**–**	–	**–**	–	**10–17**	–	**13–17**	–
Highest Viral Load in Oral Secretions	1×10^6^	–	2×10^5^	–	2×10^5^	–	–	–	6×10^7^	–	4×10^7^	–	4×10^7^	–	2×10^7^	2.5×10^8^	–	–	–	–	1.2×10^4^	–	7.8×10^4^	–

Two animal challenge studies utilized 6×10^3^ pfu; for West African MPXV this is 0.05×LD_50_ (A) and for Congo Basin MPXV 1×LD_50_ (B). Additionally a low-dose Congo Basin MPXV challenge (0.1×LD_50_) (C) was done. For each study, eight cages with holes on one side were utilized to individually cross-house eight animals. One challenged animal was cross-housed from an uninfected animal from the time of exposure of the challenged animal. Observations of disease and molecular findings are shown for primary MPXV challenged prairie dogs (bold-print) and naive prairie dogs; animals in the table are paired in the same primary challenged/naive pairs as was utilized during the study (i.e. PD8141 was housed across from PD8145). The days the sample tested positive are shown for viral DNA in blood, viral DNA in oral secretions and virus in oral secretions.

* No serum on previous two collection days;

** missing sample;

NT Not Tested.

### Congo Basin MPXV Clinical Findings and Disease Transmission

#### High dose congo basin MPXV (Table 1b)

All four animals challenged intranasally with 5×10^3^ pfu of Congo Basin MPXV developed illness characteristic of MPXV disease progression within this animal model. Disseminated skin pustules on these primary challenged animals were first observed between days 10–13. Viral DNA in blood was detected in all four animals as was viable virus from oropharyngeal swabs. Viral shedding began on day 6 (n = 2) or 10 (n = 2) p.i. and lasted until day 24 in the surviving animal. Viral loads from the oropharyngeal samples peaked at 2×10^7^–6×10^7^ pfu/mL; higher than the peak viral loads seen in oropharyngeal samples (2×10^5^–1×10^6^ pfu/mL) collected from the West African MPXV challenged animals. As we have seen in previous studies, the clinical symptoms observed in the Congo Basin MPXV challenged animals were more severe than the West African MPXV challenged prairie dogs with the same challenge dose. In addition to skin lesions, clinical symptoms included inappetence, dehydration, nasal congestion, pus/blood in mouth, labored breathing, facial edema, pus from genitals, and swollen paws (caused by large number of lesions). On days 13 and 14 p.i., three of the primary animals had to be euthanized due to extreme morbidity. Anti-OPXV seroreactivity was detected beginning on day 13 from all three of these animals by ELISA; by day 17 for the surviving animal.

Although three of the Congo Basin MPXV primary challenged animals were euthanized on days 13 or 14 p.i., these animals began shedding virus on days 6–10 and therefore all four naive animals in this arm of the study had potential exposures to infectious virus. However, only one naive/exposed animal showed signs of MPXV illness (PD8021). This animal was housed across from the primary challenged animal (PD8121) that survived infection. Naive PD8021 was observed to have skin pustules 20 days after the start of the study followed by inappetance, dehydration and diarrhea. Approximately 15 lesions were observed on this secondarily infected animal at peak infection. Viral DNA in blood was detected from PD8021from days 17 to 34 and viable virus was found in oropharyngeal samples between days 20–34 with a peak viral load of 2.5×10^8^ pfu/mL at day 31. This animal developed evidence of seroconversion to OPXV reactivity by western blot and ELISA at study end (day 34). 34 days after the start of the study, and 14 days after symptom onset, this secondarily infected animal perished due to MPXV infection.

**Figure 1:**
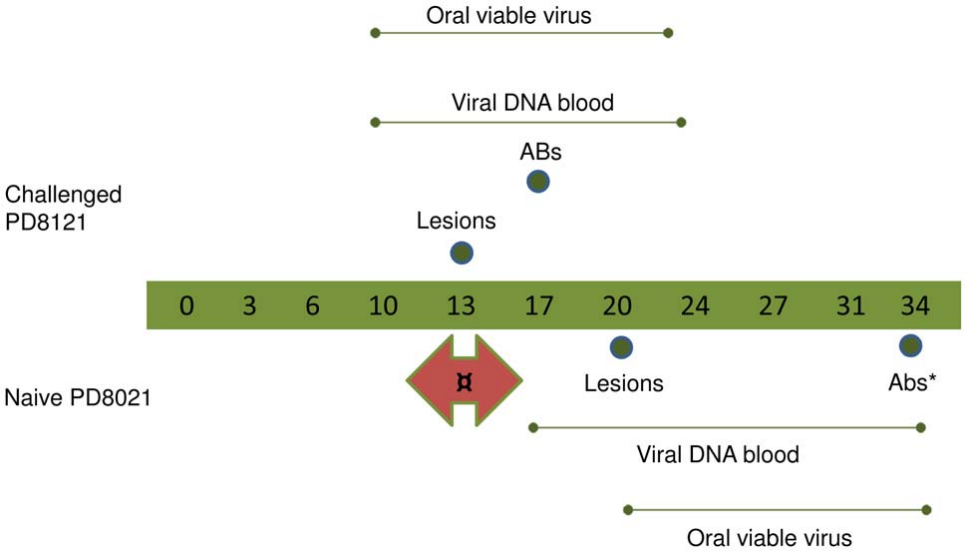
Schematic showing primary challenged and secondarily infected prairie dogs’ disease progression. Transmissability of MPXV was studied within the prairie dog MPXV model. For each study, eight cages with holes on one side were utilized to individually cross-house eight animals. Schematic shows disease and molecular findings for primary challenged animal (PD8121) and cross-housed secondarily infected animal (PD8021). Arrow indicates possible infection * 2 serum samples before day 34 not available **¤** Only used lesion presentation for estimation of infection.

The disease progression of the high dose Congo Basin MPXV challenged animal PD8121 and secondarily infected PD 8021 are depicted in Figure 1. Secondarily infected PD 8021 had a delayed but similar disease progression as the primary Congo Basin MPXV challenged animals.

#### Low dose congo basin MPXV (Table 1c)

Since three of the four animals challenged with 5×10^3^ Congo Basin MPXV had to be euthanized due to extreme morbidity, and the surviving primary challenged animal did transmit the virus to the naive animal, we did a follow up study with a lower challenge dose (7×10^2^) of Congo Basin MPXV. Since prior studies have suggested a steep dose response curve and the LD_50_ value for this animal model is 5.9×10^3^ pfu, we anticipated little to no death with a challenge dose of 7×10^2^. Additionally, this lower-dose Congo Basin MPXV challenge (0.1×LD_50_) was more similar to the LD_50_ utilized in the West African MPXV challenge arm of the study (0.05×LD_50_).

Of the four animals challenged with a low-dose Congo Basin MPXV inoculum, only two showed symptomatic illness (PDs 9049 and 9105); PD9049 was euthanized on day 21 due to extreme weight loss (25%). Skin pustules on these two animals were observed on days 10 and 13 p.i. Viral DNA was detectable from these two primary challenged animals between days 6–20 and 13–17. Viral shedding from oropharyngeal samples occurred between days 10–17 and 13–17 with peak loads reaching 1.2×10^4^–7.8×10^4^. In addition to skin lesions, inappetence was noted for both primary challenged animals. PD 9049 was additionally observed to have labored breathing while under anesthesia on day 14 p.i. By day 21 p.i., both animals were recovering from the infection. However, PD 9049 reached the 25% weight loss and therefore humane euthanasia was mandated. PDs 9105 and 9049 were positive for anti-OPXV antibodies by ELISA beginning on day 13. The other two challenged animals (PDs 9125 and 9034) did not develop any signs or symptoms of MPXV infection nor illness during the course of the study (i.e. lesions, viral shedding, viral DNAemia); at euthanasia on day 35 no evidence of any OPXV seroreactivity by ELISA or Western blot was found.

Of the two naive animals (PDs 9126 and 9088) cross housed from the two animals with overt MPXV illness, neither animal showed any clinical symptoms during the study (inappetence, skin lesions, lethargy, etc.). Furthermore, testing of all oral swabs and blood samples from the naive animals taken during the study were negative for viral DNA, as was sera for reactivity to OPXVes via Western blot and ELISA, at study end (day 35).

### Immunological Evidence of MPXV Exposure

**Figure 2:**
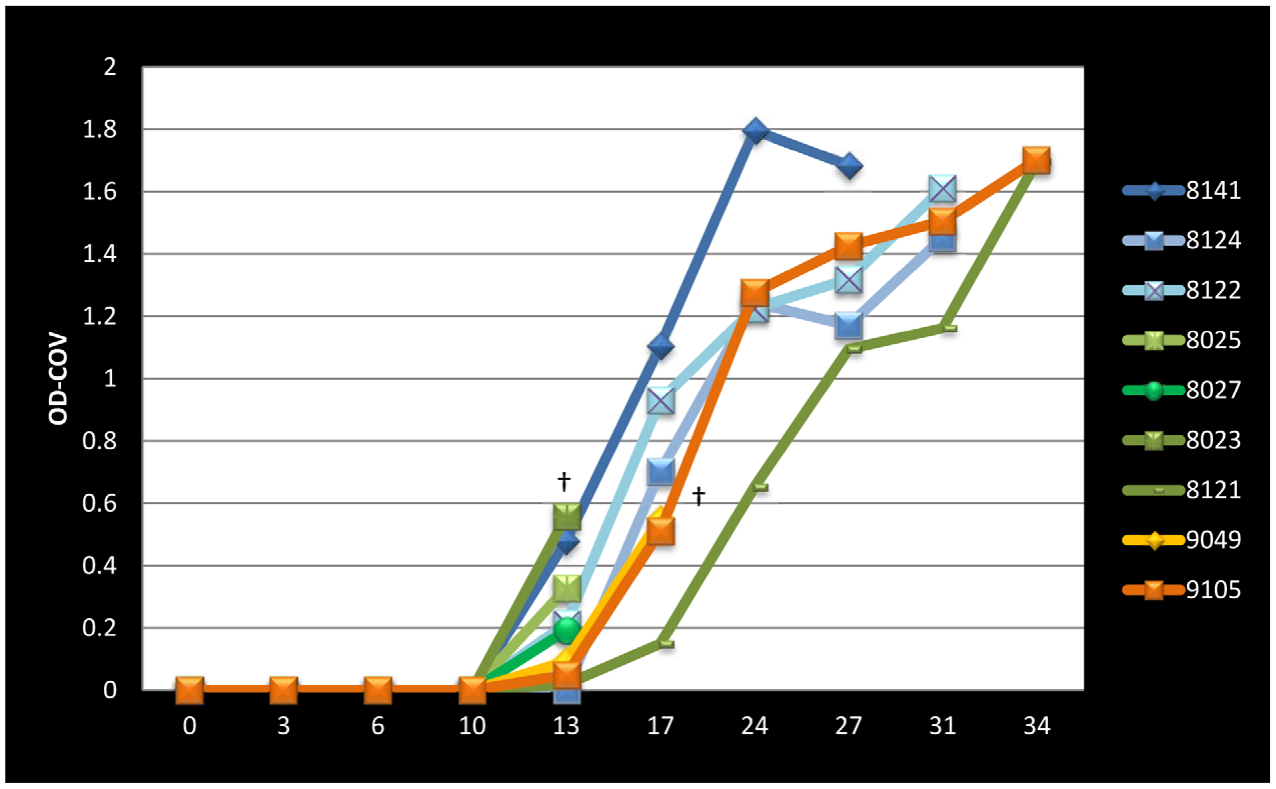
Antibody Response of Primary Infected Prairie dogs. West African MPXV primary challenge prairie dogs (n = 3) are shown in blue lines. High dose Congo Basin MPXV primary challenged prairie dogs (n = 4) are shown in green lines (PDs 8141, 8124 and 8122 died on day 13 as indicated by cross). Low dose Congo Basin MPXV primary challenged prairie dogs (n = 2) are shown in orange lines (PD9049 died on day on day 21 as indicated by cross).

For those prairie dogs that were challenged with MPXV and developed infection and symptomatic illness, antibodies against OPXVes were detectable by ELISA beginning by days 13–17 for all animals (Fig 2). The naive animal (PD8021) that became infected during the study and subsequently died had detectable anti-OPXV antibodies by day 34 (serum samples from the previous two collection days were not obtained due to dehydration). All of the other naive prairie dogs, as well as those primary challenged animals that did not develop overt symptoms of illness were negative for antibodies against OPXVes by ELISA.

**Figure 3:**
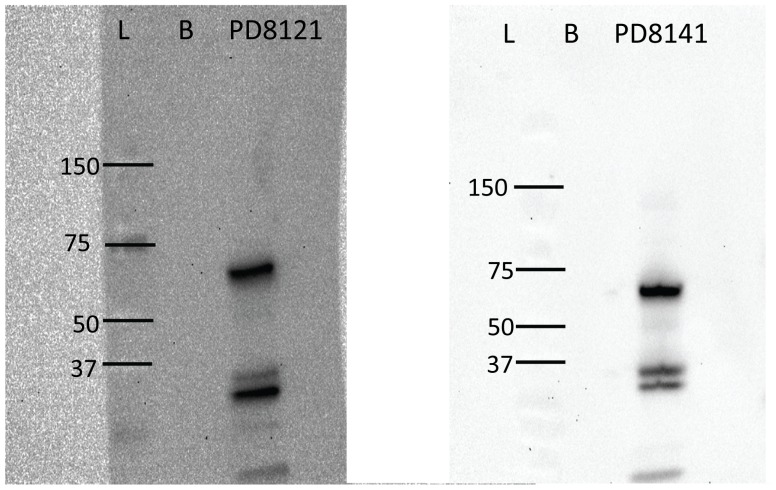
Western Blot of prairie dog sera. Sera from animals was incubated with semi-purified MPXV using standard Western Blot techniques. Results from sera collected from two representative MPXV infected priairie dogs (PD8121 and PD8141) are shown. L = molecular ladder; B = BSC40 cell lysate.

Because testing of serum from all primary challenged/symptomatically ill prairie dogs was positive by ELISA, Western blot was only done on representative (n = 5/9) primary challenged animals for better characterization of the immunological response during a MPXV infection (Table 1 panels a, b and c; Figure 3). From these representative sera, three bands were consistently seen in all primary-challenged animals. An immunodominant doublet was seen in the range of 30–39 kDa. The third immunodominant band measured 62 kDa in primary challenged animals. Serum from the naive/exposed without overt symptoms as well as experimentally infected but apparently uninfected prairie dogs produced no bands, supporting evidence that the proteins detected in sera from primary challenged animals were due to reactions between anti-MPXV antibodies and viral antigens, and that the naive animals without overt symptoms of disease had not been infected with MPXV.

### Blood Chemistry Values

**Figure 4:**
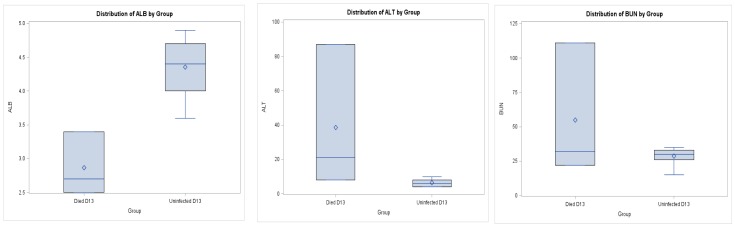
Blood chemistry values for prairie dogs suggesting impaired liver function . Values shown for albumin (ALB), alanine aminotransferase (ALT) and blood urea nitrogen (BUN) values including the average (diamond), median (line) and the interquartile range indicated by the box (25th-75th percentiles); the lines extend to minimum and maximum values. Values are shown from three animals challenged with high dose Congo Basin MPXV that died on day 13 compared to values from uninfected animals on day 13.

Blood chemistry values were compared between the three animals that died in the high-dose Congo Basin challenge at day 13 post-infection to those values from uninfected animals on day 13. The only value that was significantly changed in the animals that died were increased globulin (GLOB) values (p = 0.04) which may be associated with production of immunoglobulins within these animals (Data not shown). However trends were observed in liver chemistry values including decreased albumin (ALB), and increased alanine aminotransferase (ALT) and blood urea nitrogen (BUN) suggesting those animals that died from infection had impaired liver function (Fig 4).

### Necropsy Findings in Animals that Died During MPXV Infection

#### Molecular findings

**Figure 5:**
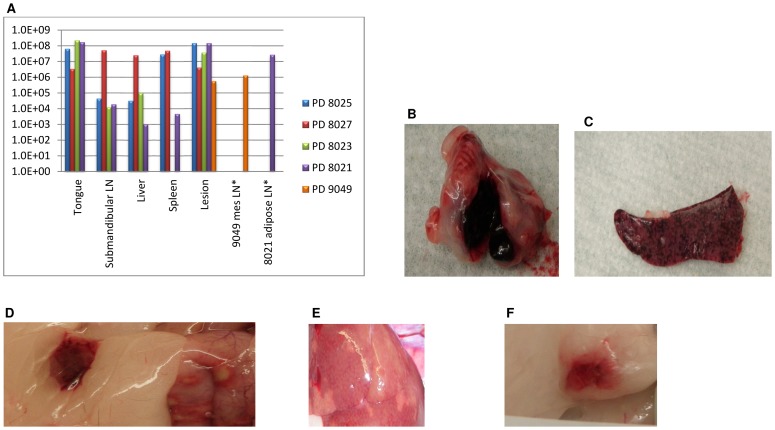
Necropsy Findings from MPXV infected prairie dogs. Transmissibility of MPXV was studied within the prairie dog MPXV model. For each study, eight cages with holes on one side were utilized to individually cross-house eight animals. One challenged animal was cross-housed with an uninfected animal from the time of exposure of the challenged animal. Loads of virus within tissues harvested from animals that died due to MPXV infection are shown in pfu/g on a log scale (A). Gross pathological findings included necrotic/hemorrhagic submandibular lymph nodes (B); characteristic mottled spleen seen during MPXV infections (C); enlarged LN in abdominal adipose tissue and pox lesions visible in intestinal tissue (D); discolored liver with multifocal areas of paleness (E); and enlarged mesentery lymph nodes (F). Asterisks (*) indicate tissues that were only taken from one animal.

All postmortem tissues tested from those animals that died or were euthanized due to infection were positive for viral DNA. The three animals challenged with 5×10^3^ pfu Congo Basin MPXV (PDs 8025, 8027, 8023) had high loads of viable virus in all tissues tested; with the exception of the spleen sample from PD 8023 (plaques were not visible in the first two wells due to the monolayer being destroyed) (Fig 5a). Similarly, the naive animal that became infected and subsequently died during the higher dose Congo Basin study (PD 8021) also had high levels of virus within all tested tissues (Fig 5a). This is in contrast to the low-dose Congo Basin MPXV challenged animal (PD 9049) that was euthanized later in infection due to weight loss; presumably during the recovery phase. This animal did not have viable virus in the tongue, submandibular lymph nodes, liver or spleen; however, virus was recovered from the mesentery lymph node tissues and lesion sample of this animal (mesentery lymph node tissue was not taken from the other animals). Those tissues listed that had cleared infection within PD 9049 are considered early targets of MXPV infection, while mesentery lymph nodes and lesion tissues have been shown to become infected later during a systemic infection (Hutson et al, manuscript in preparation).

#### Gross findings in animals that died or were euthanized

The three PDs 8025, 8027 and 8023 that were challenged with 5×10^3^ pfu Congo Basin MPXV and euthanized on day 13 or 14 p.i. had similar gross pathological findings. Lymphadenopathy was a common finding; the submandibular lymph nodes appeared black, hemorrhagic and enlarged (Fig 5b). The liver and spleen tended to be friable and had a multifocally mottled appearance (Fig 5c). In one of these animals, the kidneys had a white, mottled appearance. The naive animal that became infected with Congo Basin MPXV (PD 8021) and subsequently died from infection 34 days after the start of the study had similarly enlarged, necrotic submandibular lymph nodes and an enlarged, discolored liver. Additionally, the intestines were heavily affected by the infection; hemorrhagic areas were observed in the small intestines, large intestine and enlarged, blood engorged lymph nodes were present in the adipose tissue. MPXV lesions were observed along the intestinal tract (Fig 5d) as well as on the lung. The uterus/ovaries of this animal also had hemorrhagic foci. PD 9049 was the animal that was euthanized in the low-dose Congo Basin MPXV challenge towards the end of infection. The lymphadenopathy in this animal was slight and was not associated with necrosis or hemorrhage. As seen in the other animals that died during infection, the liver (Fig 5e) and spleen had mottled appearances. The kidneys were friable and there was a hemorrhagic lymph node associated with this organ that was attached to the body wall. The mesenteric lymph nodes were moderately enlarged and hemorrhagic in PD9049 (Fig 5f). In contrast, the mesenteric lymph nodes were normal in animals that were euthanized or died at an earlier time-point after experimental infection. Although PD 9049 exhibited sustained weight loss, there was feed material seen in the stomach during necropsy. No gross lesions were found in other organs in the affected animals.

#### Histology

**Figure 6:**
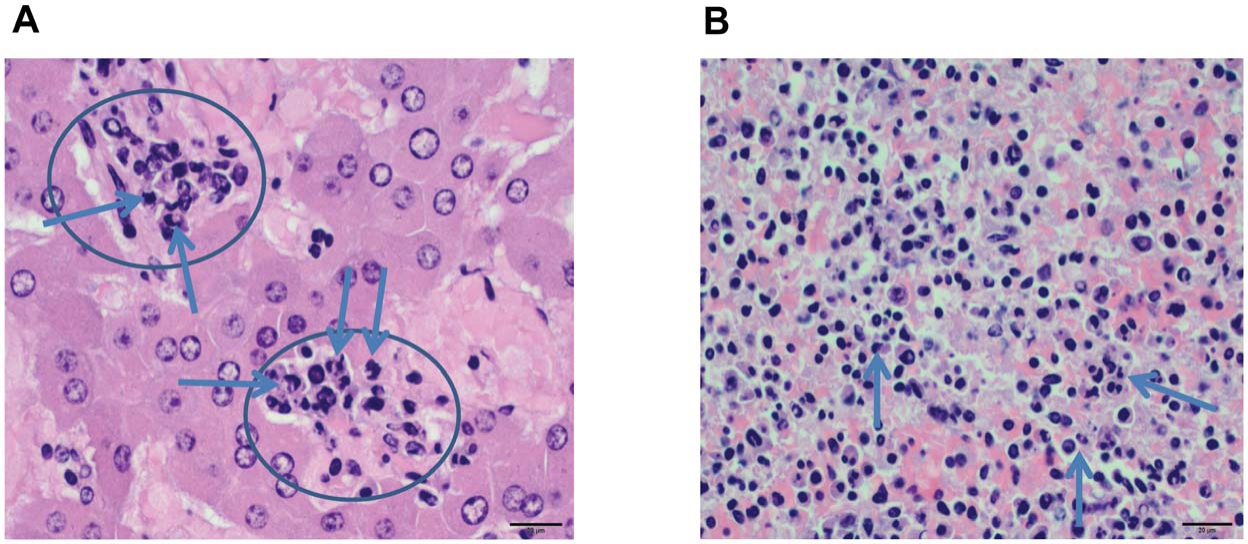
H&E stained liver and spleen from a prairie dog that died due to MPXV infection (PD8021). 6a shows a high magnification photomicrograph of the liver (400× original magnification, scale bar = 20 micrometer). There are two foci (circles) composed of necrotic inflammatory cells and hepatocytes within the image. Necrotic cells are highlighted (arrows). Figure 6b shows a high magnification photomicrograph of the red pulp of the spleen (400× original magnification, scale bar = 20 micrometer). There are scattered foci with necrotic cells (arrows) in the red pulp.

**Figure 7:**
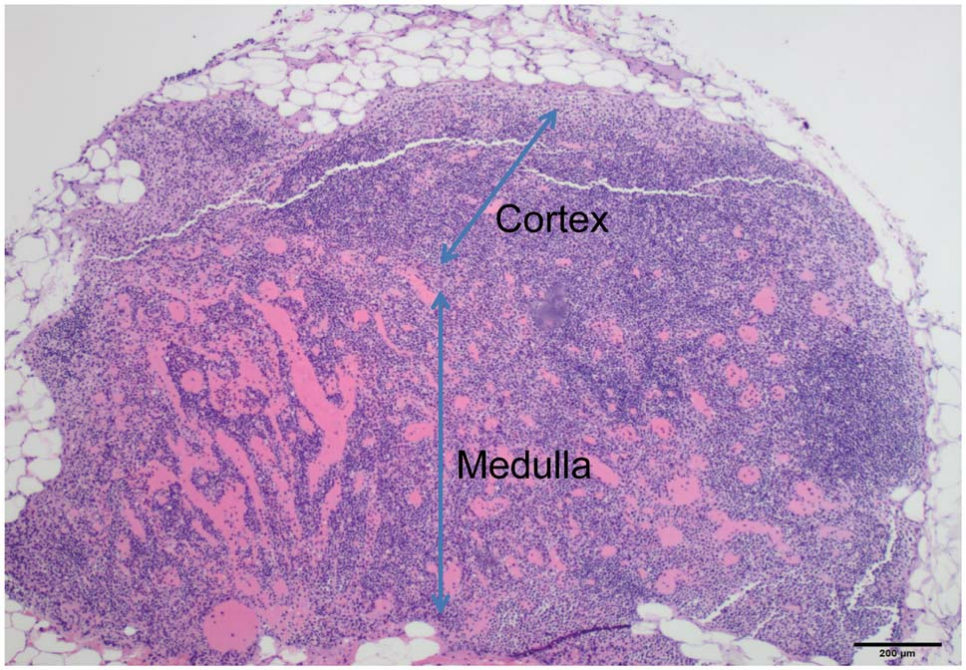
H&E stained mesenteric lymph node collected from a prairie dog that died due to MPXV infection (PD8021). Low magnification photomicrograph of mesenteric lymph node embedded in mesenteric adipose tissue (40× original magnification, scale bar = 200 micrometer). The cortical lymphoid tissue is moderately depleted and the medullary sinuses are engorged with blood.

**Figure 8:**
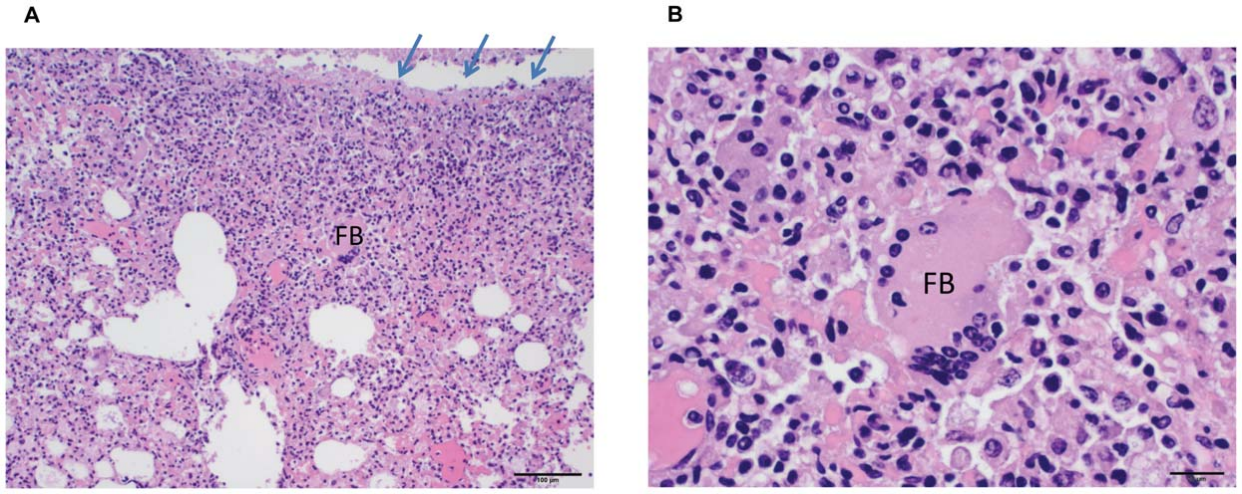
H&E stained lung tissue collected from a prairie dog that died due to MPXV infection (PD8021). Figure 8a shows a low magnification photomicrograph of the lung (100× original magnification, scale bar = 100 micrometer). The pleural surface (arrows) is covered with an inflammatory exudate admixed with fibrin. Underneath there is a dense inflammatory infiltrate filling the alveoli and expanding alveolar walls. In the center of the image there is a foreign body type giant cell (FB). Figure 8b shows a high magnification photomicrograph of the foreign body giant cell (FB) in the infiltrate in the lung (400× original magnification, scale bar = 20 micrometer).

**Figure 9:**
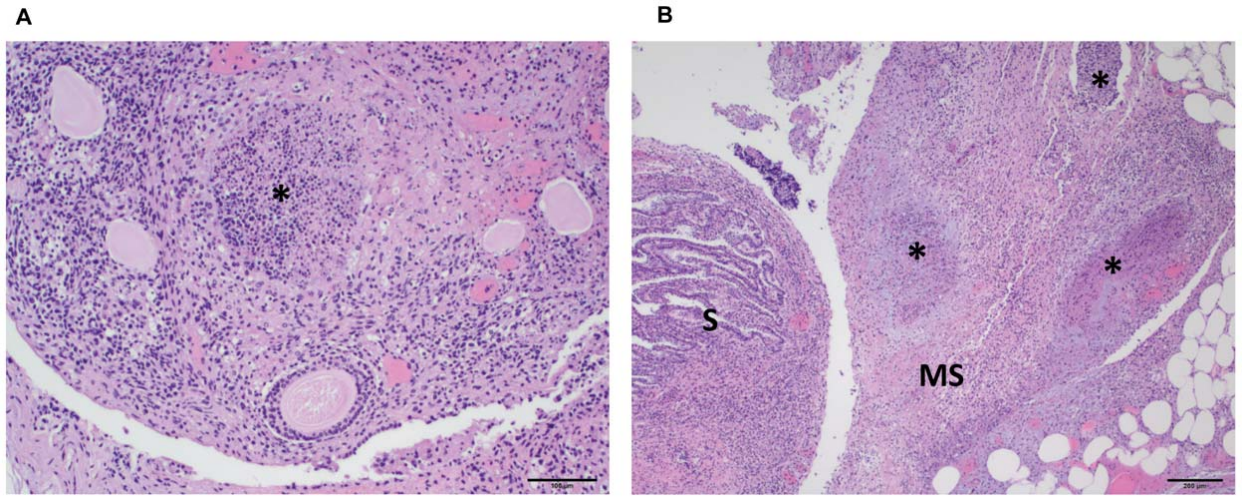
H&E stained reproductive tissues collected from a prairie dog that died due to MPXV infection (PD8021). Figure 9a shows a low magnification photomicrograph of the ovary with a necrotic focus (*) and associated inflammatory infiltrate (100× original magnification, scale bar = 100 micrometer). Figure 9b shows a low magnification photomicrograph of the salphynx (S) and adjacent mesosalphynx (MS) (40× original magnification, scale bar = 200 micrometer). There is extensive necrosis and pockets of inflammation (*) in the mesosalphynx.

Representative tissues of interest (liver, spleen, kidney, adipose tissue with associated lymph node, lung, and ovaries) from prairie dog PD 8021 were examined histologically. All tissues from this prairie dog displayed histological lesions attributable to an active MPXV virus infection. The liver had widespread small foci of acute necrosis with associated mononuclear inflammatory infiltrate (Fig 6a). Necrotic areas were characterized by loss of normal hepatic architecture and accumulation of cellular debris. Hepatocytes adjacent to necrotic areas were moderately swollen. The spleen from this animal displayed marked lymphoid depletion and numerous small foci of necrosis within the red pulp (Fig 6b). The necrotic areas were characterized by loss of normal splenic architecture and accumulation of cellular debris. The kidney had widespread tubular degeneration and multifocal necrosis. The mesenteric lymph node exhibited moderate congestion affecting the medullary sinuses, but no appreciable hemorrhage or necrosis (Fig 7). The lung had a focal necrotic area with associated fibrinous pleuritis (Fig 8a). This necrotic area was diffusely infiltrated by mononuclear inflammatory cells and rare multinucleated giant cells (Fig 8b). The ovary was multifocally necrotic and the parovarian adipose tissue also had multifocal areas of necrosis and associated mononuclear inflammatory cells (Figs 9a and 9b).

## Discussion

In the current study, transmission of the two MPXV clades was minimal via respiratory transmission. Transmission did not occur in any naive animals cage-housed at a fixed 4 inches across from West African MPXV cage-housed infected animals. Low rates of transmission occurred during Congo Basin MPXV transmission studies; 1/4 (25%) naive animals housed across from Congo Basin MPXV high-dose challenged animals developed an infection and neither of the 2 naive animals housed across from two low-dose Congo Basin MPXV challenged/infected symptomatically ill animals became infected. Compared to prior studies, based on these findings from a small sample set, respiratory transmission appears to be less efficient than contact as a mechanism of transmission within this model.

These findings are in contrast to a previous study we did with the prairie dog MPXV model to study transmissibility potential of the West African clade MXPV [14]. In the previous study, when naive animals were housed in cages across from MPXV infected animals similar in methods as the current study; 100% of the naive animals were infected with MPXV. However, the cages were placed closer together (approximately one inch apart) and were not separated by a metal rod as in the current study; therefore the cages could have shifted and become in close enough proximity for animals to touch noses. Therefore, one hypothesis which warranted testing was whether the animals in the previous study were able to touch noses and exchange nasal discharge (previously shown to be virus-laden). Based on the findings from the current study in which none of the naive animals housed across from West African MPXV infected prairie dogs became infected, we can hypothesize that animals in the previous study were indeed able to exchange nasal discharge due to the close proximity of the cages. Based on the comparison of these two studies, we have demonstrated that transmission of West African MPXV is only likely to occur if animals are in very close proximity/direct contact with one another. However, one of our Congo Basin MPXV high-dose challenged animals in this study did transmit the virus to a naive/exposed animal which became infected and subsequently died. This difference in rate of transmission and increased disease burden in secondarily infected animals between the two MPXV clades could be attributed to the greater viral shedding in Congo Basin MPXV infected animals; these animals excreted 1–2 logs higher loads of virus than West African MXPV infected animals challenged with the same inoculation dose. Previous studies with this animal model also showed higher loads of viable virus from samples collected from Congo Basin MXPV infected prairie dogs compared to West African MXPV challenged animals [11], [15].

Only three of the four animals challenged with 5×10^3^ pfu of West African MPXV became infected and only two of the four challenged with low-dose Congo Basin MPXV became infected. There are several possible explanations for why these animals were not infected after experimental inoculation. Although the animals are completely anesthetized during inoculation, it is possible that the virus was not efficiently delivered via intranasal challenge. We have previously seen a steep dose response in this animal model, even half a log lower than the intended viral inoculation could result in little or no disease. This steep dose response was noted in a previous study in which 6×10^3^ pfu of each MPXV clade resulted in overt MPXV disease in all four animals [15]. However, in the same study, approximately one log lower dose of 6×10^2^ pfu resulted in only one of the four challenged animals becoming infected for each clade. If the intended inoculation was efficiently given, another possibility is that this animal’s innate immune system was able to effectively clear the virus before the adaptive immune system became involved. Because these are wild/outbred animals, we do observe differences in disease response and progression.

One of the major limitations of the current study is the small number of animals utilized. Although we were able to show animal to animal transmission of MPXV within this and previous studies, additional respiratory transmission studies with MPXV would be worthwhile. The described prairie dog MPXV animal model will be useful to study anti-virals or therapeutics that may stop/decrease transmission of the virus. However, to truly understand the transmissibility potential of the two MPXV clades, studies that evaluate aerosol/particle numbers and size will be of benefit [18]. Such controlled studies that are able to generate different particle sizes as well as different transmission scenarios (i.e. coughing and breathing) to assess the spread of MPXV aerosols would be very informative in understanding the transmission potential of MPXV amongst potential animal reservoirs as well as within human populations.

All four of the animals challenged with 5×10^3^ pfu (high-dose) of Congo Basin MPXV developed signs and symptoms of characteristic MPXV illness including disseminated skin pustules 10–13 days p.i., upper airway disease, anti-OPXV antibodies and viral shedding. However, three of these animals were euthanized due to extreme morbidity on days 13 or 14 p.i. Of the four naive animals housed across from the Congo Basin MPXV challenged animals, only the naive animal housed across from the challenged animal that survived infection developed characteristic MPXV illness. Skin lesions on this secondarily infected animal were first observed 20 days after the start of the study; a maximum number of 15 lesions were counted at peak infection. This animal developed a measurable antibody titer and began shedding virus from oropharyngeal samples on day 20, and perished due to infection 34 days from study initiation. Because three of the four Congo Basin MXPV primary challenged animals were euthanized during the course of infection, we did a follow-up study with a lower challenge dosage of Congo Basin MPXV to see if the transmission rate would have been higher had the primary challenged animals survived infection. Only two of the animals challenged with 7×10^2^ pfu of Congo Basin MPXV developed infection, similar to results we have seen previously with a comparable challenge dose [15]; neither of the two naive animals housed across from the two infected animals showed evidence of MPXV infection. If we combine the animals successfully primarily infected with Congo Basin MPXV from each challenge dose (n = 6), we observed evidence of exposure to, and infection with MPXV in 1/6 (16.6%) of the naive animals. Infection, resulting in illness, was transmitted if exposure was to a symptomatically ill animal that was shedding virus for a period greater than seven days.

In this and previous studies with the prairie dog MPXV model, we observe the development of disseminated lesions on animals between days 10–13 p.i. and the detection of OPXV antibodies between days 13–17 [11], [14], [15]. We can therefore use this data to hypothesize when the naive animal (PD8021) was likely exposed to MPXV. The secondarily infected animal that was infected by Congo Basin MPXV was probably exposed between days 11–14 p.i. based on lesion presentation (initial time of antibody production was not used as we were not able to collect serum from this animal on two consecutive days). Since peak viral shedding from the oral cavity occurs around day 13 from those animals experimentally challenged, the 11–14 day hypothesized timeframe of MPXV exposure would be conceivable.

Western Blot of serum samples from representative primary challenged animals (Table 1) were screened for immunodominant bands. Three bands were consistently seen in all of these primary-challenged animals (Fig. 3). One immunodominant doublet was seen in the range of 30–39 kDa. This homologous doublet is consistent with two immune-dominant proteins characterized in vaccinia virus infections (a closely related OPXV): the 32 kDa D8 envelop protein, and the 39 kDa A4 core protein [19]. The third immunodominant band measured 62 kDa in primary challenged animals and is consistent with the molecular weight of A10, a well-characterized vaccinia virus core protein [19]. These three immunodominant bands were also observed in another study which utilized MPXV infected prairie dog sera as positive controls [20]. Serum from all of the naive/uninfected prairie dogs produced no bands, supporting the conclusion that proteins detected in sera from primary challenged animals were due to reactions between anti-MPXV antibodies and viral antigens and that the naive animals without overt symptoms were not exposed to MPXV during the course of the study. The primary challenged animals without overt signs of disease were also negative for bands by Western blot, supporting the belief that these animals were not infected.

Animals that died due to MPXV infection had high loads of virus within all tested tissues taken at necropsy; with the exception of PD 9049 that was euthanized later in infection. This animal was observed to be recovering from infection, but euthanasia was mandated due to weight loss. However, the molecular data and gross pathology indicated that this animal was in the final stages of viral clearance. Unlike the other animals that died or were euthanized at peak infection, this animal did not have enlarged/necrotic submandibular lymph nodes, but did have enlarged blood-engorged mesentery lymph nodes. In a previous study we have seen that the submandibular lymph nodes are associated with early replication of the virus within this animal model, while the mesentery lymph nodes are involved later during infection, probably during a second replication of the virus (Hutson et al, manuscript in preparation). Within all of the animals that died, extensive gross pathological changes were observed in numerous organs including liver, spleen, lymph nodes, intestines and the uterus/ovaries. Pox lesions were also observed on several internal tissues, both by gross pathology as well as histological examination of tissues from one of these animals. The liver had pathological changes observed in all of the animals that died during this study, and these observations were supported by blood chemistry results. The trends of increased hepatic enzyme ALT and blood urea nitrogen (BUN) seen on day 13 within the animals that died during infection suggest decreased liver function. Infection with MPXV in a ground squirrel model also reported impaired blood chemistry levels; including ALP and ALT values [21]. However in primate challenge studies with MPXV, these blood chemistry values were not reported to differ from uninfected animals [22], [23]. In our current study, the albumin levels were low in the three animals that died from infection compared to uninfected animals, also suggestive of liver disease. Hypoalbuminemia was also a common finding in humans infected with MPXV; occurring in 50% of patients [24].

Evidence has been given to support differences in transmissibility of the two MPXV clades within humans [10], [25]. The West African clade has been documented to rarely transmit human to human; during the U.S. outbreak with the West African clade, no human to human cases were seen [25]. However, the Congo Basin clade of MPXV has had up to six sequential transmissions of virus from human to human documented [10]. The increased transmissibility of Congo Basin MXPV within a human setting is one of the noted differences between the two viral strains [7]. Although disease transmission was minimal during our study, apparent respiratory transmissibility of the Congo Basin clade virus was slightly greater than that of the West African MXPV clade virus (16.7% and 0% respectively) within the prairie dog MPXV model. In the current study, the increased transmissibility of the Congo Basin MPXV clade by respiratory route compared to the West African clade may be due to greater respiratory shedding of Congo Basin MXPV. Based on these findings, respiratory transmission of MPXV appears to be less efficient than close or direct contact within the prairie dog MPXV model.

### Disclaimer

The findings and conclusions in this report are those of the author(s) and do not necessarily represent the official position of the Centers for Disease Control and Prevention.
