# Correction: Transmissibility of the Monkeypox Virus Clades via Respiratory Transmission: Investigation Using the Prairie Dog-Monkeypox Virus Challenge System

**DOI:** 10.1371/annotation/18ebd26a-21ef-43ff-b20d-c22f433e5714

**Published:** 2014-01-03

**Authors:** Christina L. Hutson, Nadia Gallardo-Romero, Darin S. Carroll, Cody Clemmons, Johanna S. Salzer, Tamas Nagy, Christine M. Hughes, Victoria A. Olson, Kevin L. Karem, Inger K. Damon

There were multiple errors in the legend for Figure 2. The correct legend is: 

"Transmissibility of MPXV was studied within the prairie dog MPXV model. For each study, eight cages with holes on one side were utilized to individually cross-house eight animals. One challenged animal was cross-housed with an uninfected animal from the time of exposure of the challenged animal. West African MPXV primary challenge prairie dogs (n=3) are shown in blue line. High dose Congo Basin MPXV primary challenged prairie dogs (n=4) are shown in green lines (PDs 8025, 8027 and 8023 died on day 13/14 as indicated by cross). Low dose Congo Basin MPXV primary challenged prairie dogs (n=2) are shown in orange lines (PD9049 died on day on day 21 as indicated by cross)."

